# Molecular characteristics of microsatellite stable early-onset colorectal cancer as predictors of prognosis and immunotherapeutic response

**DOI:** 10.1038/s41698-023-00414-8

**Published:** 2023-07-01

**Authors:** Can Lu, Xiaopeng Zhang, Josefine Schardey, Ulrich Wirth, Kathrin Heinrich, Luca Massiminio, Giulia Martina Cavestro, Jens Neumann, Alexandr V. Bazhin, Jens Werner, Florian Kühn

**Affiliations:** 1 Department of General, Visceral, and Transplant Surgery, Ludwig-Maximilians-University Munich, 81377 Munich, Germany; 2 Department of Colorectal Surgery and Oncology, Key Laboratory of Cancer Prevention and Intervention (Ministry of Education), The Second Affiliated Hospital, School of Medicine, Zhejiang University, Hangzhou, China; 3 Zhejiang Provincial Clinical Research Center for CANCER & Cancer Center of Zhejiang University, Hangzhou, China; 4 Key Laboratory of Carcinogenesis and Translational Research (Ministry of Education/Beijing), Gastrointestinal Cancer Center, Peking University Cancer Hospital and Institute, Beijing, China; 5 Institute of Laboratory Medicine, University Hospital of LMU Munich, Munich, Germany; 6 Department of Medicine III, University Hospital, Ludwig-Maximilians-University Munich, 81377 Munich, Germany; 7 Experimental Gastroenterology Laboratory, Gastroenterology and Endoscopy Department, San Raffaele Scientific Institute, Milano, Italy; 8 Institute of Pathology, Medical Faculty, Ludwig-Maximilians-University Munich, 81377 Munich, Germany; 9 German Cancer Consortium (DKTK), Partner Site Munich, 81377 Munich, Germany; 10 Bavarian Cancer Research Center (BZKF), Partner Site Munich, Munich, Germany

**Keywords:** Colorectal cancer, Cancer genomics

## Abstract

The incidence of early-onset colorectal cancer (EO-CRC, in patients younger than 50) is increasing worldwide. The specific gene signatures in EO-CRC patients are largely unknown. Since EO-CRC with microsatellite instability is frequently associated with Lynch syndrome, we aimed to comprehensively characterize the tumor microenvironment (TME) and gene expression profiles of EO-CRC with microsatellite stable (MSS-EO-CRC). Here, we demonstrated that MSS-EO-CRC has a similar pattern of tumor-infiltrating immune cells, immunotherapeutic responses, consensus molecular subtypes, and prognosis as late-onset CRC with MSS (MSS-LO-CRC). 133 differential expressed genes were identified as unique gene signatures of MSS-EO-CRC. Moreover, we established a risk score, which was positively associated with PD-L1 expression and could reflect both the level of tumor-infiltrating immune cells and the prognosis of MSS-EO-CRC patients. Application of this score on the anti-PD-L1 treatment cohort demonstrated that the low-risk score group has significant therapeutic advantages and clinical benefits. In addition, candidate driver genes were identified in the different-sidedness of MSS-EO-CRC patients. Altogether, MSS-EO-CRC exhibits distinct molecular profiles that differ from MSS-LO-CRC even though they have a similar TME characterization and survival pattern. Our risk score appears to be robust enough to predict prognosis and immunotherapeutic response and therefore could help to optimize the treatment of MSS-EO-CRC.

## Introduction

Colorectal cancer (CRC) is the third most diagnosed carcinoma and the second leading cause of cancer-associated mortality globally^1^. Although the incidence of late-onset CRC (LO-CRC) diagnosed in patients 50 years or older has steadily declined over the last two decades in most Western countries^2^, the cases of early-onset CRC (EO-CRC) diagnosed in those younger than 50 years have increased alarmingly worldwide. By the 2030s, it is estimated that EO-CRC will account for one-quarter of rectal cancers and 10 to 12% of colon cancers^2,3^. So far, the underlying causes for the rising trends of EO-CRC are unknown, but early-life exposures, Western-style diet, microbial dysbiosis, and physical inactivity might contribute to the expansion of the EO-CRC population^4,5^.

Accumulating studies reported that EO-CRCs tend to have a more advanced TNM (tumor-node-metastasis) stage, higher prevalence of left-sided carcinoma and poorly differentiated tumors, a higher proportion of microsatellite instability-high (MSI-H) status and more germline mutations compared to LO-CRCs^5,6^. Extensive efforts have been made to characterize the somatic mutational profiling of EO-CRCs^7,8^, which failed to discover previously unknown alterations to elucidate the pathogenesis of these carcinomas or to guide clinical therapy. However, the unique transcriptional features in EO-CRCs remain elusive. Increasing evidence indicates that most EO-CRCs with MSI-H are associated with Lynch syndrome^9,10^. Regarding the well-known germline mutations of mismatch repair (MMR) genes in Lynch syndrome, we intend to identify the potential molecular mechanism for developing EO-CRCs with microsatellite stable (MSS-EO-CRC).

Primary tumor location is essential in predicting the prognosis and the drug responses for CRC patients^11^. Clinically, right-sided and left-sided CRCs are divided according to proximity to the splenic flexure. Although different sidedness of CRC represents the distinction in the mutational spectrum and molecular expression patterns^12^, the biological effects of tumor location on MSS-EO-CRC patients remain unclear.

In the present study, multiple transcriptional profiles were systematically integrated to evaluate the characteristic features of MSS-EO-CRC compared to LO-CRC with MSS (MSS-LO-CRC). Based on the differentially expressed genes in MSS-EO-CRC, we constructed a risk score that significantly correlated with the tumor microenvironment (TME) characterization and showed a promising potential to predict response to anti-programmed death-ligand 1 (PD-L1) immunotherapy. Furthermore, we depicted the genetic variants and endogenous growth factor receptor (EGFR)-related molecules’ expression between different sidedness of MSS-EO-CRC.

## Results

### Characterization of tumor microenvironment and prognosis of MSS-EO-CRC patients

**Fig. 1:**
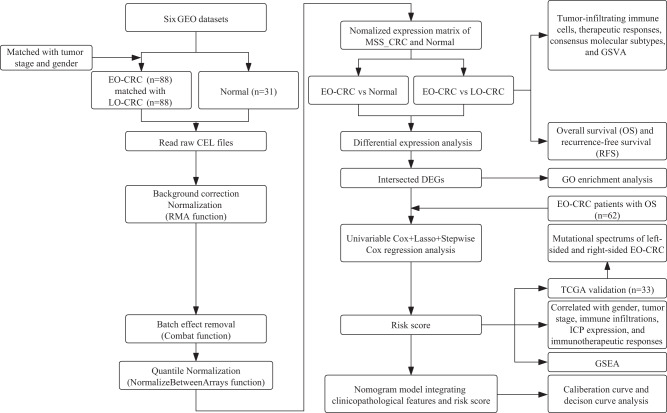
Analysis flow diagram of the study. EO-CRC and LO-CRC patients are from the cohort of CRC with MSS. GEO Gene Expression Omnibus, EO-CRC early-onset colorectal cancer, LO-CRC late-onset colorectal cancer, MSS_CRC colorectal cancer with microsatellite stable, GSVA gene set variation analysis, DEGs differentially expressed genes, GO gene ontology, TCGA The Cancer Genome Atlas, ICP immune checkpoint, GSEA gene set enrichment analysis.

**Fig. 2:**
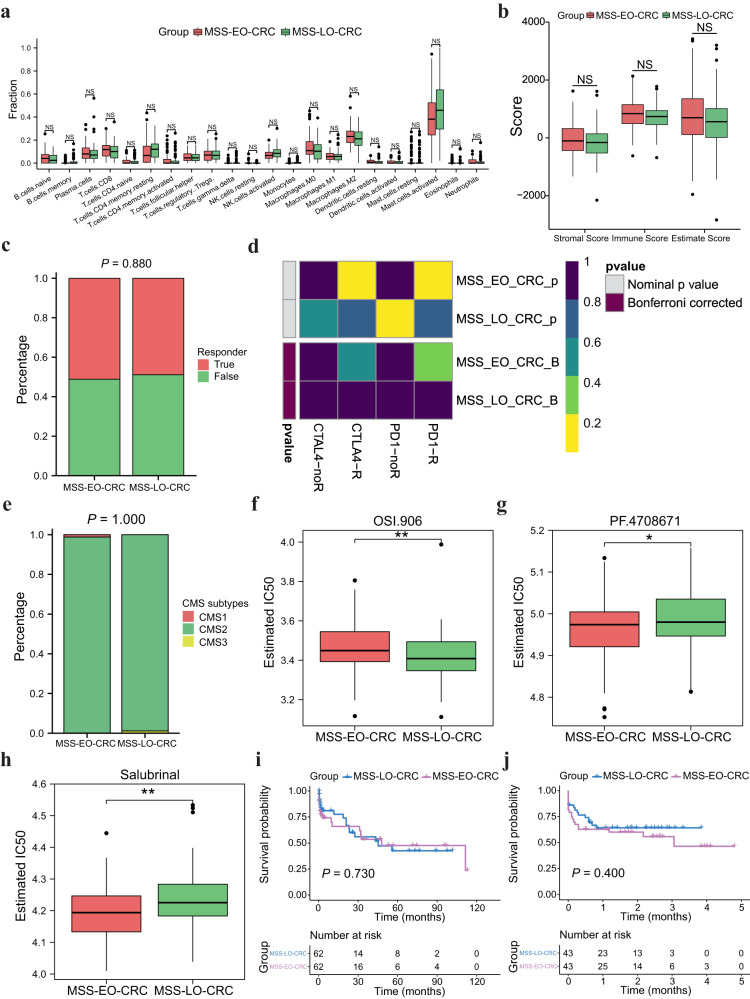
Characterization of tumor microenvironment and prognosis of early-onset CRC with MSS. **a** Comparison of tumor-infiltrating immune cells between MSS-EO-CRC and MSS-LO-CRC. *P*-values were corrected using the Benjamini–Hochberg method. **b** Comparison of the enrichment score between MSS-EO-CRC and MSS-LO-CRC. **c**, **d** Comparison of Immunotherapeutic responses between MSS-EO-CRC and MSS-LO-CRC using TIDE and SubMap algorithm, respectively. **e** Consensus molecular subtype analysis of MSS-EO-CRC versus MSS-LO-CRC. **f**–**h** Drug sensitivities comparison between MSS-EO-CRC and MSS-LO-CRC. **i** Overall survival comparison between MSS-EO-CRC and MSS-LO-CRC. **j** Recurrence-free survival comparison between MSS-EO-CRC and MSS-LO-CRC. In the boxplot, the upper and lower boundaries represented the first and third quartiles, respectively, the central line signified the median and the whiskers extended to the most distant data points not considered as outliers (within 1.5 times the interquartile range). Outliers were displayed as points above and below the box-and-whisker diagram. The log-rank test *P* values were shown for each Kaplan–Meier plot. ***P < 0.001; **P < 0.01; *P < 0.05. MSS-EO-CRC early-onset colorectal cancer with microsatellite stable, MSS-LO-CRC late-onset colorectal cancer with microsatellite stable, NS no significance.

The study flowchart is depicted in Fig. 1. To exclude the potential effects of confounding variables, we matched MSS-EO-CRC patients with MSS-LO-CRC ones according to gender and tumor stage. Supplementary Table 1 summarizes the clinicopathological characteristics of 88 MSS-EO-CRC patients and 88 MSS-LO-CRC ones. The immune system is widely recognized as a critical factor determining the development and progression of CRC^13,14^. We found that MSS-EO-CRC patients displayed a similar distribution of 22 tumor-infiltrated immune subsets to MSS-LO-CRC ones (Fig. 2a). Meanwhile, no differences between MSS-EO-CRC and MSS-LO-CRC patients were detected in the overall stromal and immune components in the TME (Fig. 2b). We also conducted TIDE and Submap algorithm to predict the treatment response of the population to immunotherapy. These two cohorts have a similar treatment response rate to anti-PD1 or anti-CTLA4 drugs (Fig. 2c, d). Consensus molecular subtypes (CMSs) hold a promising role in deciphering the intrinsic heterogeneity of CRC at the gene expression level^15^, playing a crucial role in predicting a patient’s prognosis and treatment responses^16^. Our results showed that MSS-EO-CRC patients have a similar composition of CMSs as MSS-LO-CRC ones (Fig. 2e). To identify the potential drugs having different sensitivity in subgroup patients, we predicted that MSS-LO-CRC patients were more sensitive to OSI.906, while MSS-EO-CRC ones were more sensitive to PF.4708671 and Salubrinal (Fig. 2f–h). We found that MSS-EO-CRC patients have a similar survival rate to MSS-LO-CRC in overall survival (OS) and recurrence-free survival (RFS) (Fig. 2i, j).

Furthermore, we matched 33 MSS-EO-CRC with 33 MSS-LO-CRC from TCGA cohort to confirm the above findings. The clinical characteristics of these patients are listed in Supplementary Table 2. Supplementary Fig. 1 depicted that an independent CRC dataset could get the similar results as Fig. 2, except from the sensitivity difference of some drugs.

### Identification of unique gene signatures in MSS-EO-CRC

**Fig. 3:**
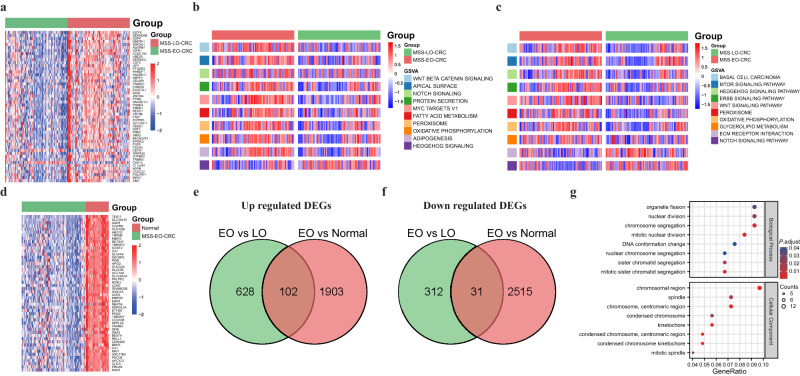
Identification of unique gene signatures in early-onset CRC. **a** The heatmap of the top 50 DEGs in MSS-EO-CRC compared to MSS-LO-CRC. **b** Heatmap showing the hall markers differences between MSS-EO-CRC and MSS-LO-CRC. **c** Heatmap showing the biological pathway differences between MSS-EO-CRC and MSS-LO-CRC. **d** The heatmap of the top 50 DEGs in MSS-EO-CRC compared to normal samples. **e**, **f** Up-regulated and down-regulated DEGs, specifically in MSS-EO-CRC, respectively. **g** GO enrichment analysis of unique gene signatures in MSS-EO-CRC. MSS-EO-CRC early-onset colorectal cancer with microsatellite stable, MSS-LO-CRC late-onset colorectal cancer with microsatellite stable, DEGs differentially expressed genes.

To identify the genetic features in MSS-EO-CRC patients, we performed a sequential analysis by comparing the gene expression matrix of MSS-EO-CRC with MSS-LO-CRC and with normal samples. Firstly, we identified 1073 differentially expressed genes (DEGs) in MSS-EO-CRC patients compared to MSS-LO-CRC, including 730 up-regulated and 343 down-regulated genes (Fig. 3a). Functional annotations based on the Gene Set Variation Analysis (GSVA) algorithm showed that these two cohorts displayed significant differences in the enriched hall marker and molecular pathways (Fig. 3b, c). MSS-EO-CRC showed higher enrichment of Wnt beta-catenin signaling, protein secretion, and metabolic activities, whereas MSS-LO-CRC displayed more potent activity in hedgehog signaling. Furthermore, the mTOR signaling pathway, Wnt signaling pathway, and metabolic pathways are markedly enriched in MSS-EO-CRC, and MSS-LO-CRC significantly enriched the extracellular matrix (ECM) receptor interaction pathway. Secondly, 4551 DEGs were obtained from the differential expression analysis between MSS-EO-CRC samples and normal controls (Fig. 3d). Considering the particular entity of EO-CRC in terms of age and carcinoma, we performed the intersection of DEGs between MSS-EO-CRC versus MSS-LO-CRC and MSS-EO-CRC versus normal to identify the genes featured in MSS-EO-CRC patients. In total, 133 DEGs consisting of 102 up-regulated genes and 31 down-regulated genes were identified as the common DEGs (Fig. 3e, f). Furthermore, the Gene Ontology (GO) enrichment analysis showed that these genes were significantly related to the mitosis activities of chromosomes and DNA (Fig. 3g). Detailed results of this functional enrichment analysis are shown in Supplementary Table 3.

### Development of the risk score for MSS-EO-CRC patients

**Fig. 4:**
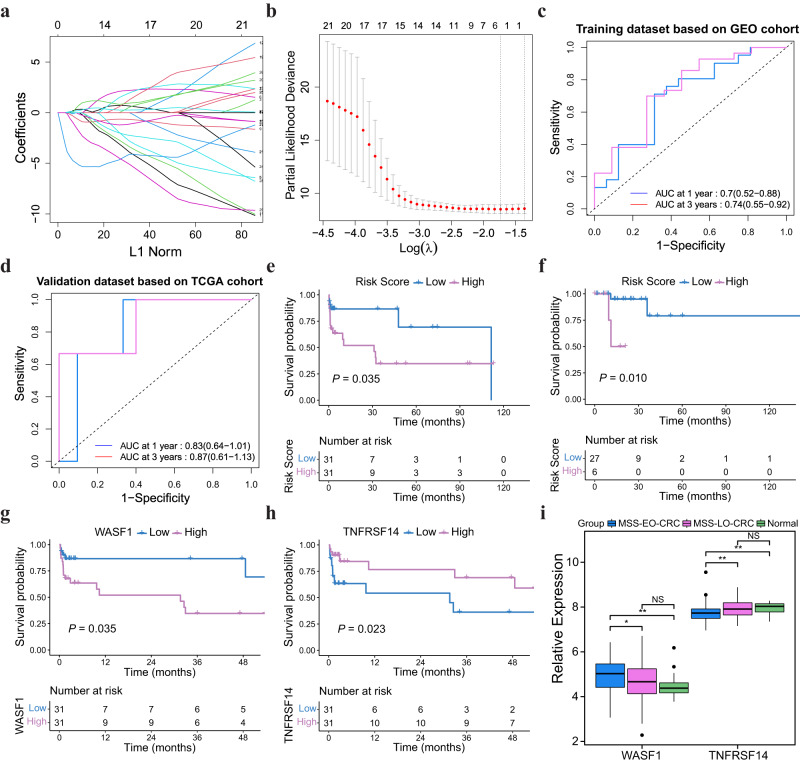
Construction of risk score and validation. **a** LASSO coefficient of the 29 genes. **b** The 10-fold cross-validation for variable selection in the LASSO model. The dotted line shows the optimal lambda values by using the minimum criteria. **c**, **d** Time-dependent ROC analyses of the risk score in the training and validation sets, respectively. **e**, **f** Kaplan–Meier plot of the risk score in the training and validation sets, respectively. **g**, **h** Kaplan–Meier plot of WASF1 and TNFRSF14 in the training set, respectively. **i** Expression of WASF1 and TNFRSF14 among MSS-EO-CRC, MSS-LO-CRC, and Normal. The upper and lower boundaries represented the first and third quartiles, respectively, while the central line signified the median. The whiskers extended to the most distant data points not considered as outliers (within 1.5 times the interquartile range), and outliers were displayed as points above and below the box-and-whisker diagram. The log-rank test *P* values were shown for each Kaplan–Meier plot. ***P < 0.001; **P < 0.01; *P < 0.05. ROC receiver operating characteristic, AUC area under the curve, MSS-EO-CRC early-onset colorectal cancer with microsatellite stable, MSS-LO-CRC late-onset colorectal cancer with microsatellite stable, NS no significance.

Since MSS-EO-CRC has a different transcriptomic landscape than MSS-LO-CRC, it is promising to construct a prognostic model for the subgroup of CRC patients regarding the potential age discrepancies. However, OS information was only available for partial MSS-EO-CRC patients. Thus, MSS-EO-CRC from GEO datasets (*N* = 62) and TCGA cohort (*N* = 33) were separately considered as training and external validation datasets to build the clinical model. The baseline clinical characteristics are summarized in Supplementary Table 4. Twenty-nine genes were identified from the training set using the univariate Cox regression analysis on the genes featured in MSS-EO-CRC (Supplementary Table 5). To refine the parameters incorporated into the model, we subsequently utilized the Least Absolute Shrinkage and Selection Operator (Lasso) Cox regression to select the substantial genes highly predictive of the OS (Fig. 4a). Three genes were identified with the lambda of 0.176 (Fig. 4b). Further, they entered a stepwise Cox regression model using a bidirectional selection strategy. Finally, WASF1 and TNFRSF14 were chosen to construct a prognostic model using a logistic regression algorithm. A risk score for prognosis prediction was determined as follows: risk score = (16.04519 × *Expr*_WASF1_) + (0.00002 × *Expr*_TNFRSF14_). We used the time-dependent ROC curves to evaluate the prognostic capacity of this risk score. The area under the curves (AUCs) for 1 year and 3 years OS were 0.70 and 0.74 for the training set (Fig. 4c), 0.83 and 0.87 for the external validation set (Fig. 4d), respectively. A risk score of 42.021 and 60.298 was separately defined as the optimal cut-off value to divide the population of training and validation set into a high-risk group and a low-risk group. Patients in the high-risk groups have significantly worse OSs than the low-risk ones in these two cohorts (*P* < 0.05) (Fig. 4e, f). Moreover, the prognostic capacity of the risk score remained robust in the subgroup analysis stratified by the tumor stage (*P* < 0.05) (Supplementary Fig. 2a, b). In addition, the distribution of gender and tumor stage were similar between high- and low-risk groups of MSS-EO-CRC patients (Supplementary Fig. 2c), which indicated that these two factors have no association with the risk score.

Furthermore, the two genes incorporated into the risk score were prognostic factors for the OS of MSS-EO-CRC patients (Fig. 4g, h), in which the expression level of WASF1 was inversely correlated with the prognosis of patients, whereas patients with higher levels of TNFRSF14 have a better prognosis than lower ones. Compared to MSS-LO-CRC and normal, WASF1 and TNFRSF14 were specifically up-regulated and down-regulated in MSS-EO-CRC, respectively (Fig. 4i). However, no differences were detected between early-stage and advanced MSS-EO-CRC in the expression of these two genes (Supplementary Fig. 2d, e).

### Characterization of the tumor microenvironment and immunotherapeutic responses in high and low-risk score groups

**Fig. 5:**
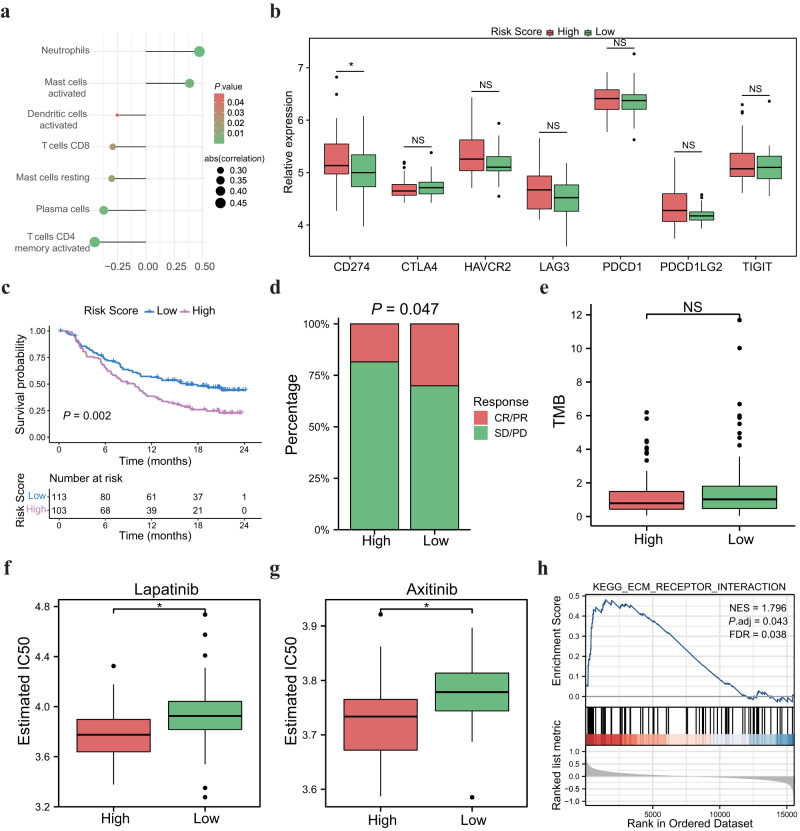
Comparison of the tumor microenvironment and immunotherapeutic responses between high and low-risk score groups based on the GEO datasets. **a** Correlation analysis between tumor-infiltrating immune cells and the risk score. **b** Expression difference of immune checkpoint between high and low-risk score cohorts. **c** Survival curve showing the low-risk score group had a better prognosis than the high-risk score group. The log-rank test *P* value were shown on the plot. **d** The proportion of patients with clinical response to anti-PD-L1 treatment in high or low-risk score groups. SD, stable disease; PD, progressive disease; CR, complete response; PR, partial response. **e** Differences in the neoantigen burden between high and low-risk score groups. **f**, **g** Distribution of estimated IC50 of lapatinib and axitinib between high and low-risk score groups, respectively. **h** GSEA result of the risk score. In the boxplot, the upper and lower boundaries represented the first and third quartiles, respectively, the central line signified the median and the whiskers extended to the most distant data points not considered as outliers (within 1.5 times the interquartile range). Outliers were displayed as points above and below the box-and-whisker diagram. ***P < 0.001; **P < 0.01; *P < 0.05. GSEA gene set expression analysis, NS no significance.

The immune infiltration played a critical role in regulating the development and progression of CRC via conducting pro-tumor or anti-tumor biological effects. We found that the risk score was negatively correlated with the infiltration level of CD8^+^ T cells, activated memory CD4^+^ T cells, and activated dendritic cells in the TME of MSS-EO-CRC (Fig. 5a). Since the ICP could significantly alter the function of T lymphocytes, we evaluated the relationship between the risk score and the expression level of seven ICP-related molecules in MSS-EO-CRC patients. Our study proved that the high-risk group has a markedly higher CD274 (PD-L1) level than the low-risk one (Fig. 5b), suggesting the risk score may correlate with the response to immunotherapy. Thus, we evaluated the capacity of the risk score in predicting the treatment response to anti-PD-L1 antibody Atezolizumab using the IMvigor210 immunotherapy cohort. Patients with high-risk scores had a worse survival rate than patients with low-risk scores (Fig. 5c). The percentage of patients who responded to the anti-PD-L1 drug in the high-risk score group was remarkably lower compared to the low-risk score group (Fig. 5d). However, no difference of neoantigen was detected between high- and low-risk score groups (Fig. 5e).

Targeted therapy has become a promising strategy for advanced-stage cases. Identifying subgroups of patients more sensitive to certain drugs is critical to provide individualized therapy. Our study suggested that the low-risk score group was more sensitive to two tyrosine kinase inhibitors, lapatinib and axitinib, than the high-risk one (Fig. 5f, g). Moreover, we applied the Gene Set Enrichment Analysis (GSEA) analysis on GEO datasets to decipher the molecular mechanism underlying the risk score. As shown in Fig. 5h, the ECM receptor interaction pathway was significantly enriched in the tumors of the low-risk score group.

### Construction and assessment of a predictive nomogram

**Fig. 6:**
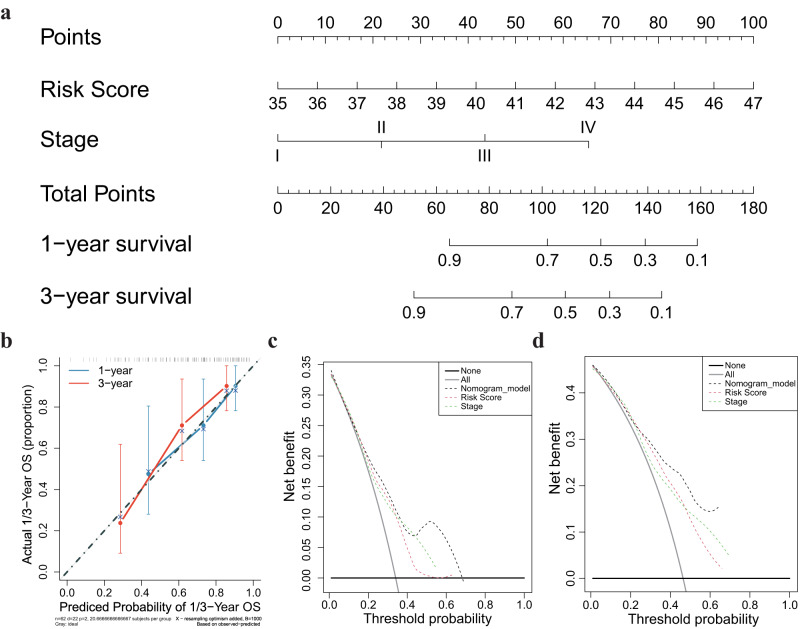
Nomogram construction and validation. **a** A nomogram predicting survival rate at one and three-year after surgery for MSS-EO-CRC patients. **b** Calibration curves for the nomogram. **c**, **d** DCA curves depicting the comparison between the nomogram and either risk score or tumor stage for predicting 1- and 3-year overall survival in MSS-EO-CRC, respectively. MSS-EO-CRC early-onset colorectal cancer with microsatellite stable, DCA decision curve analysis.

We performed univariate and multivariate Cox regression analyses on MSS-EO-CRC patients from GEO datasets to assess the risk score, tumor stage, age, and gender as independent prognostic markers. The tumor stage and risk score were identified as the independent prognostic factors for the GEO datasets (tumor stage, HR: 2.05; 95% CI: 1.24–3.38; *P* < 0.01; risk score, HR: 1.32; 95%: 1.06–1.63; *P* < 0.05). We provided the details in Supplementary Table 6. Thus, we integrated the risk score and tumor stage into a nomogram model to maximally increase the predicted probability on 1-year and 3-year OS (Fig. 6a). According to the goodness of fit between the predicted survival probability and actual survival rate on calibration plots, the nomogram has a better prediction on short-term survival (1-year) than long-term survival (3-year) (Fig. 6b). In addition, the nomogram has a higher concordance index (C-index) (0.702, 95% CI: 0.640–0.764) than either tumor stage (0.650, 95% CI: 0.587–0.713) or the risk score (0.649, 95% CI: 0.588–0.710) alone. Decision curve analysis (DCA) demonstrated that the nomogram model has the most significant net benefit for MSS-EO-CRC patients compared to the rest two factors (Fig. 6c, d).

### Mutational spectrums and EGFR-related molecular expression in different sidedness of MSS-EO-CRC patients

**Fig. 7:**
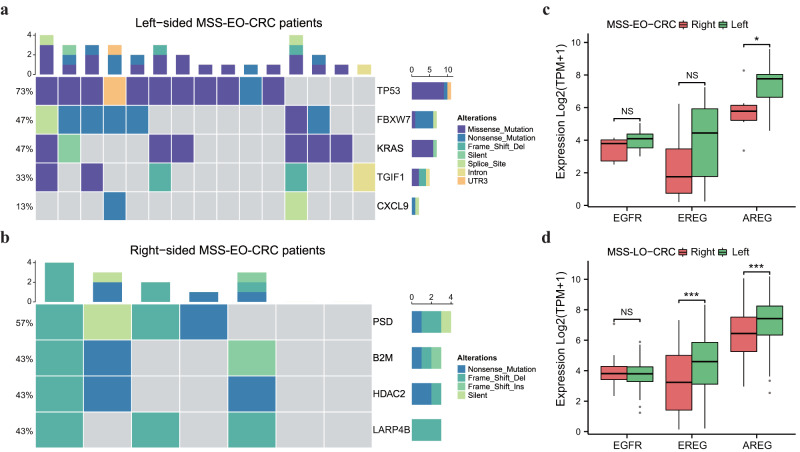
Mutational spectrums and EGFR-related molecular expression in different sidedness of early-onset CRC. **a**, **b** Mutational spectrums of left-sided and right-sided MSS-EO-CRC patients, respectively. **c**, **d** Expression of EGFR-related molecules in MSS-EO-CRC and MSS-LO-CRC, respectively. In the boxplot, the upper and lower boundaries represented the first and third quartiles, respectively, the central line denoted the median and the whiskers extended to the most distant data points not considered as outliers (within 1.5 times the interquartile range). Outliers were displayed as points above and below the box-and-whisker diagram. ***P < 0.001; **P < 0.01; *P < 0.05. MSS-EO-CRC early-onset colorectal cancer with microsatellite stable, MSS-LO-CRC late-onset colorectal cancer with microsatellite stable, NS no significance.

Due to the distinctive molecular characteristics of CRC with different sidedness, we intend to explore the potential effects of tumor location on the mutational landscape and genetic expression of MSS-EO-CRC. Based on the somatic mutation data of MSS-EO-CRC patients from TCGA, we predicted the candidate driving genes using the MutSigCV algorithm with a *p*-value less than 0.001. As displayed in Fig. 7a, five genes have been identified as the significantly mutated genes (SMGs) for left-sided MSS-EO-CRC patients, including TP53, FBXW7, KRAS, TGIF1, and CXCL9. Meanwhile, PSD, B2M, HDAC2, and LARP4B might act as the driving genes for the tumorigenesis of right-sided MSS-EO-CRC patients (Fig. 7b).

In addition, multiple studies indicated that right-sided CRC has significantly higher expression of EGFR and its ligands than left-sided ones^12,17,18^. According to the available information on tumor location and MSS status from TCGA, we separately selected 25 MSS-EO-CRC and 285 MSS-LO-CRC patients to evaluate the expression pattern of the above molecules. As is depicted in Fig. 7c, d, MSS-EO-CRC has similar expression changes of AREG with MSS-LO-CRC, whereas EREG has distinct expression characteristics in different sidedness of MSS-LO-CRC but MSS-EO-CRC patients.

## Discussion

The rising trend of EO-CRC will impose an immersive socioeconomic burden in a modern-aged society. To reduce the incidence of EO-CRC, the underlying biological mechanism for the tumorigenesis of EO-CRC could provide novel insights to hinder the development of CRC in individuals younger than 50 years of age. Based on tumor stage and gender, we matched MSS-EO-CRC patients with MSS-LO-CRC ones to comprehensively characterize the TME and gene expression patterns of MSS-EO-CRC. Furthermore, a risk score was built to predict the prognosis and immunotherapeutic treatment response of MSS-EO-CRC patients.

We first demonstrated that MSS-EO-CRC patients have a similar composition of tumor-infiltrating immune cells and stromal components with MSS-LO-CRC ones. This finding is in line with data published by Ugai et al., which showed a comparable proportion of nine subsets of T cells, three subtypes of macrophages, and eight subgroups of myeloid cells between MSS-EO-CRC and MSS-LO-CRC patients^19^. Meanwhile, these two CRC cohorts also have identical response rates to ICP inhibitors. Since only CRC patients with MSS were included in our study, most of these subjects are refractory to immune monotherapy, mainly caused by the low levels of tumor-infiltrating lymphocytes and tumor mutation burden^20,21^. According to the classification of CMSs, our study displayed that CMS2 was the dominant molecular subtype for both MSS-EO-CRC and MSS-LO-CRC patients. In contrast, one clinical study indicated that EO-CRC has a comparable composition of CMS2 with LO-CRC, but also explicitly showed CMS1 was the most common subtype in EO-CRC^22^. This discrepancy is primarily due to the distinct inclusion criteria for MSS status. Furthermore, we proved that MSS-EO-CRC patients have a similar OS and RFS as MSS-LO-CRC, which aligns with multiple studies^23–25^. Therefore, MSS-EO-CRC patients have a similar TME landscape and comparable survival with MSS-LO-CRC ones.

Comprehensively illustrating the enriched pathways can give us more insights into the different potential mechanisms of tumorigenesis between EO-CRC and LO-CRC. EO-CRC patients have more robust Wnt signaling activation than LO-CRC^26^. Consistently, our study depicted that the mTOR signaling pathway and Wnt signaling pathway might play a more significant role in promoting the progression of MSS-EO-CRC than counterparts. It is widely known that Wnt and mTOR pathways play a critical role in promoting the progression of cancer. Therefore, MSS-EO-CRC patients may be more sensitive to Wnt or mTOR-targeting drugs than MSS-LO-CRC ones. In addition, 133 DEGs were identified as the unique gene signatures for the MSS-EO-CRC cohort. The biological enrichment analysis depicted that these genes were involved with the cellular mitosis of cancer cells. Previous studies reported that loss of mitosis regulation could lead to the carcinogenesis via the dysregulated cell cycle and aberrant proliferation^27^. Also, several mitosis-associated molecules are involved with the tumorigenesis and metastasis of cancer^28–31^, including CRC. Therefore, the identified gene sets may participate in the development and progression of MSS-EO-CRC via regulating mitosis. These results indicated that MSS-EO-CRC has distinct patterns of molecular mechanism and gene expression compared to MSS-LO-CRC.

Meanwhile, MSS-EO-CRC has the highest WASF1 expression and the lowest TNFRSF14 compared to MSS-LO-CRC and controls. Our study also proved that WASF1 had a detrimental role in MSS-EO-CRC patients, whereas TNFRSF14 seems a protective factor. WASF1, also known as WAVE1, could activate the actin-related protein 2/3 complex, causing actin polymerization^32^. Due to the actin cytoskeleton’s critical role in mediating cancer cell migration to the blood or lymphatic system^33,34^, WASF1 has an essential role in cancer metastasis and invasion. Many studies reported that the down-regulation of WASF1 could significantly inhibit the progression and invasion of prostate cancer and ovarian cancer^35,36^ and promote anti-drug-induced apoptosis of leukemia cells^37,38^. It has been reported that WASF1 depletion could decrease the proliferative and invasive ability of epithelial ovarian cancer (EOC) via the PI3K/AKT and p38/MAPK signaling pathways^35^. Also, elevated expression of WAVE1 is associated with a worse prognosis in EOC^39^, which is in line with our findings. However, no available studies depicted the biological function of WASF1 in CRC. TNFRSF14, also known as tumor necrosis factor receptor superfamily 14, encodes the receptor HVEM activating either co-stimulatory or co-inhibitory signaling pathways on immune cells^40,41^. It is expressed in lymphocytes and myeloid lineage cells and highly expressed in endothelial cells and adipocytes^42^. TNFRSF14/BTLA has the similar inhibitory effect with PD-L1/PD-1 to attenuate the activation of T helper cells^43^. Recently, increasing studies indicated the functional activity of TNFRSF14 in cancer^44–46^. Boice et al. found that it could oppose lymphoma development via the inhibitory cell-cell interactions with BTLA^44^. In bladder cancer, the knockdown of TNFRSF14 significantly enhanced the proliferation of bladder cancer cells through the activation of the Wnt/β-catenin-dependent pathway^46^. Conflicting results have been reported on the prognostic effect of TNFRSF14 on cancer patients; increased expression of TNFRSF14 was correlated with worse OS in chronic lymphocytic leukemia and clear cell renal cell carcinoma^45,47^, whereas the opposite correlation was observed in breast cancer and bladder cancer^48,49^. Interestingly, our study indicated that MSS-EO-CRC patients with higher TNFRSF14 expression have better OS than lower ones. Hence, WASF1 and TNFRSF14 have the potential to participate in the development and progression of MSS-EO-CRC.

To our knowledge, this is the first study proposing a prognostic model based on gene expression profiles for MSS-EO-CRC patients. Our risk score was associated with tumor-infiltrated immune cells of MSS-EO-CRC and had a reliable prediction on the prognosis of patients. It is widely recognized that tumors with the infiltration of T cells and PD-L1 expression in the parenchymal are more likely to acquire clinical responses to ICP inhibitors^50^. Here, the risk score has been demonstrated to reflect such features of TME in MSS-EO-CRC. By applying this score to the anti-PD-L1 treatment cohort of metastatic urothelial cancer, we found that the low-risk score group was associated with the immune-inflamed phenotype and higher infiltration of CD8 + T cells, thus, better prognosis and a higher response rate. Besides, the nomogram model was constructed based on the risk score and tumor stage to predict the survival of MSS-EO-CRC patients. We also proved that this model could provide a more reliable prediction than either risk score or tumor stage alone. Consequently, the risk score and the nomogram model could contribute to evaluating the prognosis and immunotherapeutic responses of MSS-EO-CRC patients.

Furthermore, many studies performed the genomic mutational comparison between EO-CRC and LO-CRC patients^7,8,51^. Even so, they failed to depict the mutational landscape in different sidedness of EO-CRC patients. In the present study, we demonstrated that five genes might act as the driver gene for left-sided MSS-EO-CRC, namely TP53, FBXW7, KRAS, TGIF1, and CXCL9. In contrast, PSD, B2M, HDAC2, and LARP4B might be involved in the development of right-sided MSS-EO-CRC. Several studies consistently pointed out that EO-CRC patients have more frequent TP53 alterations than LO-CRC^7,52,53^. Pilozzi et al. also showed that KRAS mutations have higher rates in EO-CRC than LO-CRC^54^. In addition, we found that two subtypes of CRC have nearly similar expression patterns of EGFR and its ligands except for EREG. These findings indicated that MSS-EO-CRC patients have distinct mutational spectrums in different sidedness.

The primary limitation of our study is the relatively low number of MSS-EO-CRC patients, which made us unable to construct a prognostic model for long-term survival. Meanwhile, a sizeable MSS-EO-CRC cohort is needed to further validate our model’s predictive reliability. Due to the unavailability of cell lines or animal models particularly associated with MSS-EO-CRC, we failed to assess the biological function of WASF1 and TNFRSF14 in this subgroup CRC. Although this study included a limited number of EO-CRC subjects with MSS from TCGA, we initially hinted that distinct driver genes might play a significant role in the tumorigenesis of different-sided MSS-EO-CRC. On the other hand, our study comprehensively characterized the molecular and clinical features of MSS-EO-CRC and then proposed a prognostic model to predict the patients’ survival and ICP inhibitors’ response.

MSS-EO-CRC has specific gene signatures and different patterns of tumorigenesis from MSS-LO-CRC, whereas they present a similar TME characterization and prognosis. A robust risk score and a nomogram model were established to potentially predict OS and immunotherapeutic responses of MSS-EO-CRC patients, which may contribute to identifying high-risk patients suitable for more intensive therapy.

## Materials and methods

### Data collection and processing

A comprehensive genomic analysis based on available datasets of CRC has been performed. Searching strategy (“colon” or “colorectal” or “rectal”) and (“cancer*“ or “neoplas*“ or “dysplasia”) and (“homo sapiens”) and (“gse”) was conducted on Gene Expression Omnibus (GEO) database to find all suitable CRC datasets. The eligibility criteria of GEO datasets for inclusion in our study were listed in the following (Supplementary Fig. 3): (1) Sequencing data type: transcriptional profiles; (2) Sample type: tissue; (3) Samples size: larger than 20; (4) Clinicopathological information: MSI status, age, and tumor stage. Considering the heterogeneity of GEO datasets across different platforms, a total of six GPL570 platform-based datasets (GSE39582, GSE39084, GSE9348, GSE170999, GSE18088, and GSE75316) were enrolled in this study (Supplementary Fig. 3), among which GSE39582 and GSE9348 contained corresponding normal samples, and GSE39582 and GSE39084 provided survival information. The clinical data of those datasets were downloaded from the corresponding GEO website or published literature, and the details are shown in Supplementary Table 7. Only CRC patients with MSS were recruited to exclude the known genetic effects of inherited cancer syndrome. Nearest neighbor matching based on tumor stage and gender was performed to match MSS-EO-CRC patients with MSS-LO-CRC ones for genetic and survival analysis in the ratio of 1:1 using the *MatchIt* R package^55^. The standard mean difference evaluated the matching quality before and after matching for each covariate, depicted in Supplementary Fig. 4a–c. 176 CRC patients and 31 normal controls were selected from these six GEO datasets in this study. The robust multichip average algorithm was conducted to uniformly merge the raw CEL files of the above-enrolled subjects for background correction and normalization. The combat function of the *sva* R package and the normalizeBetweenArrays function of the *limma* R package was sequentially applied to remove the batch effects and perform quantile normalization on the merged GEO dataset (Supplementary Fig. 5). The probes were annotated into gene symbols based on the GPL570 annotation files. When multiple probes matched one gene, we regarded the median of these probes as its expression value. In total, 15,620 protein-coding genes were annotated in the merged dataset. Therefore, this final GEO dataset was considered the normalized expression profiles of CRC patients and normal controls.

The Cancer Genome Atlas (TCGA) somatic mutation data were obtained using *TCGAbiolinks* R package^56^. As for gene expression profiles from the TCGA-COAD and READ cohort, the FPKM (fragment per kilobase per million) and counts data, as well as the corresponding clinical data, were downloaded from the Genomic Data Commons (GDC) data portal. Moreover, the immunohistochemistry staining determined the MSI status of these patients according to the expression of mismatch repair proteins. 33 MSS-EO-CRC patients with MSS were selected from the TCGA cohort.

Anti-PD-L1 treatment cohort derived from a multicenter, single-arm clinical trial (IMvigor210) provided the transcriptional profiles and clinical follow-up data of patients with metastatic urothelial cancer, which was used as the dataset for predicting drug responses for PD-L1 inhibitors^57^.

### Immune estimation of the TME

CIBERSORT was utilized to estimate the infiltrating level of 22 immune cells consisting of innate and adaptive immune subsets in the TME^58^. Furthermore, the ESTIMATE algorithm was applied to evaluate the enrichment score of immune and stromal components in the TME, including the immune score, stromal score, and estimate score^59^.

### CMS subtypes

The consensus molecular subtype (CMS) of the merged GEO dataset was determined using the single sample predictor implemented in the R package *CMSclassifier*^15^.

### Prediction of immunotherapy response

The Tumor Immune Dysfunction and Exclusion (TIDE) score of each sample was calculated to predict drug response to immune checkpoint (ICP) blockade by applying the TIDE algorithm to the expression profiles^60^. Also, the subclass mapping (SubMap) algorithm was utilized to predict the immunotherapy responses by identifying the common subtypes between our expression profiles and one published transcriptional dataset consisting of 47 melanoma patients who received the anti-PD1 or anti-CTLA-4 treatment^61,62^.

### Estimation of drug sensitivity

Drug sensitivity was evaluated by the predicted half maximal inhibitory concentration (IC50) based on the analysis of gene expression profiles using the R package *pRRophetic*^63^.

### Differential expression analysis

Differential expression analysis was performed to identify the DEGs in MSS-EO-CRC patients versus normal controls and MSS-EO-CRC patients versus MSS-LO-CRC ones of merged GEO datasets via the *limma* R package^64^. Any gene with a *P* value of <0.05 and |log_2_ (Fold change)| > 0.2 was considered the DEGs. Furthermore, DEGs consistently changed in the above comparisons were identified as the genes that were specifically dysregulated in MSS-EO-CRC patients.

### Gene set variation analysis

GSVA was conducted to estimate the enrichment scores of signaling pathways and hall marker gene sets using *GSVA* R package^65^. Then, differential analysis was performed to acquire the significantly enriched pathways and hall markers in each patient’s cohort. The gene sets were derived from the MSigDB database (https://www.gsea-msigdb.org)^66,67^.

### Gene Ontology analysis and Gene set enrichment analysis

GO analysis and GSEA were conducted to determine the potential biological function related to genes or prognostic model using *ClusterProfiler* R package with the *P*-value corrected by Benjamini–Hochberg method^68^. The following parameters were used for GSEA: nPerm = 1000, minGSSize = 10, and maxGSSize = 500. Adjusted *P*-value < 0.05 was regarded as significant.

### Construction of the prognostic model

To predict the OS of MSS-EO-CRC patients, we constructed a prognostic model based on the genes dysregulated in these patients. At first, univariate Cox regression of the above genes was performed to identify the prognostic genes with *p*-values less than 0.2.

Secondly, LASSO Cox regression was performed to reduce dimensionality and select the optimal parameters from the above prognostic genes. We applied ten-fold cross-validation to determine the lambda values and select the best one with the least partial likelihood of deviance. Next, the optimal genes were identified according to the selected lambda.

Thirdly, stepwise Cox regression was conducted to determine the best model choice from the above optimal genes with the bidirectional algorithm and the Akaike information criterion. Each parameter would be assigned a regression coefficient, and a risk score was generated using the following formula:

$${{\rm{Risk}}}\,{{\rm{score}}}=\mathop{\sum }\limits_{{n}=1}^{{\rm{Num}}}({{\rm{Expression}}}_{{n}}\ast {{{\rm{RC}}}}_{{n}})$$

Where Num refers to the number of genes, Expression_n_ represents the expression level of gene_n_, and RC_n_ is the regression coefficient of gene_n_.

Furthermore, univariate and multivariate cox regression were sequentially used to identify the independent prognostic factors with a *p*-value < 0.05 from four variables: age, gender, tumor stage, and risk score. Then, the nomogram model was constructed by integrating the above factors to predict the one-year and three years OS for MSS-EO-CRC patients. Moreover, the calibration curve was conducted to evaluate the goodness-of-fit of the nomogram model. DCA was performed to assess the model’s reliability by calculating the clinical net benefit for patients at each threshold probability. Besides, Harrell’s C-index were calculated to evaluate the prediction capability of our nomogram model.

*Survival* and *glmnet* R packages were used to perform the Cox regression and LASSO Cox regression analysis, respectively. We applied *Survminer* R package to select the best cut-off point for distinguishing high and low-risk score groups in this study.

### MutSigCV

The MutSigCV (version 1.4.1) algorithm was performed to determine SMGs in specified cohorts of patients^69^. Default settings were used to select the SMGs with a *p*-value < 0.001.

### Statistics and reproducibility

Correlation analysis was performed using the non-parametric Spearman method. The two-sided unpaired Wilcoxon rank-sum test or two-sided Kruskal–Wallis test were conducted to assess the statistical difference of continuous variables. We applied the Benjamini–Hochberg method to correct the *p*-values of multiple testing. The statistical difference among categorical variables was calculated using a chi-squared test. The survival difference between groups was evaluated by a log-rank test in the Kaplan–Meier plot. All analyses were done using R software (version 4.1.0) and MATLAB R2021b. *P*-value < 0.05 was regarded as statistically significant.

### Reporting summary

Further information on research design is available in the Nature Research Reporting Summary linked to this article.

## Supplementary information

REPORTING SUMMARY

Supplementary Tables and Figures

## Data Availability

GEO datasets are publicly available in the National Center for Biotechnology Information Portal (https://www.ncbi.nlm.nih.gov/geo/), including GSE39582, GSE39084, GSE9348, GSE170999, GSE18088, and GSE75316. TCGA datasets enrolled in this study are openly available in the National Cancer Institute GDC Data Portal (https://portal.gdc.cancer.gov/). TCGA data are displayed under the Project IDs “TCGA-COAD” and “TCGA-READ.” IMvigor210 dataset is openly available in *IMvigor210CoreBiologies* (http://research-pub.gene.com/IMvigor210CoreBiologies/).

## Code availability

Analyses were conducted using R (version 4.1.0) and MATLAB R2021b. The codes used to support the findings of this study is available from the corresponding author on reasonable request.
