# Supplementary material for: Molecular characteristics of microsatellite stable early-onset colorectal cancer as predictors of prognosis and immunotherapeutic response
Source: NPJ Precis Oncol. 2023 Jul 1;7:63. doi: 10.1038/s41698-023-00414-8 (PMC10314951; doi:10.1038/s41698-023-00414-8)
Supplement: Supplementary file 1 — REPORTING SUMMARY [file 41698_2023_414_MOESM1_ESM.pdf]

nature portfolio

Corresponding author(s): Florian Kühn
Last updated by author(s): Jun 7, 2023

Reporting Summary

Nature Portfolio wishes to improve the reproducibility of the work that we publish. This form provides structure for consistency and transparency in reporting. For further information on Nature Portfolio policies, see our Editorial Policies and the Editorial Policy Checklist.

Statistics

For all statistical analyses, confirm that the following items are present in the figure legend, table legend, main text, or Methods section.

n/a	Confirmed	
☐	☒	The exact sample size (*n*) for each experimental group/condition, given as a discrete number and unit of measurement
☐	☒	A statement on whether measurements were taken from distinct samples or whether the same sample was measured repeatedly
☐	☒	The statistical test(s) used AND whether they are one- or two-sided<br>*Only common tests should be described solely by name; describe more complex techniques in the Methods section.*
☐	☒	A description of all covariates tested
☐	☒	A description of any assumptions or corrections, such as tests of normality and adjustment for multiple comparisons
☐	☒	A full description of the statistical parameters including central tendency (e.g. means) or other basic estimates (e.g. regression coefficient) AND variation (e.g. standard deviation) or associated estimates of uncertainty (e.g. confidence intervals)
☐	☒	For null hypothesis testing, the test statistic (e.g. *F*, *t*, *r*) with confidence intervals, effect sizes, degrees of freedom and *P* value noted<br>*Give P values as exact values whenever suitable.*
☒	☐	For Bayesian analysis, information on the choice of priors and Markov chain Monte Carlo settings
☒	☐	For hierarchical and complex designs, identification of the appropriate level for tests and full reporting of outcomes
☐	☒	Estimates of effect sizes (e.g. Cohen's *d*, Pearson's *r*), indicating how they were calculated

Our web collection on statistics for biologists contains articles on many of the points above.

Software and code

Policy information about availability of computer code

Data collection	No custom software or codes were used to collect the data.
Data analysis	All statistical analysis and data analyses were conducted using R version 4.1.0 with publicly available packages and MATLAB R2021b. No custom software or codes were applied to perform the data analysis.

For manuscripts utilizing custom algorithms or software that are central to the research but not yet described in published literature, software must be made available to editors and reviewers. We strongly encourage code deposition in a community repository (e.g. GitHub). See the Nature Portfolio guidelines for submitting code & software for further information.

Data

Policy information about availability of data

All manuscripts must include a data availability statement. This statement should provide the following information, where applicable:

- Accession codes, unique identifiers, or web links for publicly available datasets
- A description of any restrictions on data availability
- For clinical datasets or third party data, please ensure that the statement adheres to our policy

GEO datasets are publicly available in the National Center for Biotechnology Information Portal (https://www.ncbi.nlm.nih.gov/geo/), including GSE39582, GSE39084, GSE9348, GSE170999, GSE18088, and GSE75316. TCGA datasets enrolled in this study are openly available in the National Cancer Institute GDC Data

Portal (https://portal.gdc.cancer.gov/). TCGA data are displayed under the Project IDs "TCGA-COAD" and "TCGA-READ." IMvigor210 dataset is openly available in IMvigor210CoreBiologies (http://research-pub.gene.com/IMvigor210CoreBiologies/).

Human research participants

Policy information about studies involving human research participants and Sex and Gender in Research.

Reporting on sex and gender	N/A
Population characteristics	N/A
Recruitment	N/A
Ethics oversight	N/A

Note that full information on the approval of the study protocol must also be provided in the manuscript.

Field-specific reporting

Please select the one below that is the best fit for your research. If you are not sure, read the appropriate sections before making your selection.

☒ Life sciences ☐ Behavioural & social sciences ☐ Ecological, evolutionary & environmental sciences

For a reference copy of the document with all sections, see nature.com/documents/nr-reporting-summary-flat.pdf

Life sciences study design

All studies must disclose on these points even when the disclosure is negative.

Sample size	Sample size was not predetermined in this descriptive cohort study. All eligible patients were included in this project. Prognostic model was trained on the merged GEO datasets (n=62), and TCGA cohort (n=33) was used for testing purposes.
Data exclusions	The eligibility criteria of GEO datasets for inclusion in our study were listed in the following: 1) Sequencing data type: transcriptional profiles; 2) Sample type: tissue; 3) Samples size: larger than 20; 4) Clinicopathological information: MSI status, age, and tumor stage. In addition, only CRC patients with MSS were recruited to exclude the known genetic effects of inherited cancer syndrome. Lastly, nearest neighbor matching based on tumor stage and gender was performed to match MSS-EO-CRC patients with MSS-LO-CRC ones to exclude the potential influence of these two clinical characteristics.
Replication	The prognostic model trained in the GEO datasets was externally validated in the TCGA cohort.
Randomization	The prognostic model was validated in the training set by ten-fold cross-validation.
Blinding	Blinding was not conducted for this descriptive, algorithmic model based study.

Reporting for specific materials, systems and methods

We require information from authors about some types of materials, experimental systems and methods used in many studies. Here, indicate whether each material, system or method listed is relevant to your study. If you are not sure if a list item applies to your research, read the appropriate section before selecting a response.

Materials & experimental systems

n/a	Involved in the study	
☒	☐	Antibodies
☒	☐	Eukaryotic cell lines
☒	☐	Palaeontology and archaeology
☒	☐	Animals and other organisms
☒	☐	Clinical data
☒	☐	Dual use research of concern

Methods

n/a	Involved in the study	
☒	☐	ChIP-seq
☒	☐	Flow cytometry
☒	☐	MRI-based neuroimaging
