# Supplementary material for: Molecular characteristics of microsatellite stable early-onset colorectal cancer as predictors of prognosis and immunotherapeutic response
Source: NPJ Precis Oncol. 2023 Jul 1;7:63. doi: 10.1038/s41698-023-00414-8 (PMC10314951; doi:10.1038/s41698-023-00414-8)
Supplement: Supplementary file 2 — Supplementary Tables and Figures [file 41698_2023_414_MOESM2_ESM.pdf]

Supplementary Table 1. Clinical characteristics of MSS-EO-CRC and MSS-LO-CRC patients.

Variables	EO-CRC (n = 88)	LO-CRC (n = 88)	*P*-value
Age, year[1]	41.8 ± 6.45	64.5 ± 9.79	< .001[2]
Gender			1.00[3]
Female	52 (59.09%)	52 (59.09%)	
Male	36 (40.91%)	36 (40.91%)	
Tumor Stage (AJCC 7th)			1.00[3]
I	4 (4.55%)	4 (4.55%)	
II	31 (35.23%)	31 (35.23%)	
III	34 (38.64%)	34 (38.64%)	
IV	19 (21.59%)	19 (21.59%)	

Abbreviations: MSS-EO-CRC, early-onset colorectal cancer with microsatellite stable; MSS-LO-CRC, late-onset colorectal cancer with microsatellite stable; AJCC, American Joint Committee on Cancer.

Data were represented as n (%) unless otherwise annotated. Age[1] was presented as mean ± standard deviation.

[2] represented the Wilcoxon rank-sum test. [3] denoted the chi-squared test.

Supplementary Table 2. Clinical characteristics of MSS-EO-CRC and MSS-LO-CRC from TCGA cohort.

Variables	MSS-EO-CRC (n=33)	MSS-LO-CRC (n=33)	*P*-value
Age, year[1]	43.94 ± 4.79	72.45 ± 10.54	< .001[2]
Gender			1.00[3]
Female	18 (54.55%)	18 (54.55%)	
Male	15 (45.45%)	15 (45.45%)	
Tumor stage (AJCC 7th)			1.00[3]
I	5 (15.15%)	5 (15.15%)	
II	9 (27.27%)	9 (27.27%)	
III	13 (39.39%)	13 (39.39%)	
IV	6 (18.18%)	6 (18.18%)	

Abbreviations: MSS-EO-CRC, early-onset colorectal cancer with microsatellite stable; MSS-LO-CRC, late-onset colorectal cancer with microsatellite stable; AJCC, American Joint Committee on Cancer.

Data were represented as n (%) unless otherwise annotated. Age[1] was presented as mean ± standard deviation.

[2] represented the Wilcoxon rank-sum test. [3] denoted the chi-squared test.

Supplementary Table 3. GO enrichment analysis of unique gene signatures of MSS-EO-CRC.

Category	Term	Description	Count	*P*-value[1]	FDR
BP	GO:0048285	Organelle Fission	11	3.72E-02	3.40E-02
BP	GO:0000280	Nuclear Division	11	2.34E-02	2.14E-02
BP	GO:0007059	Chromosome Segregation	11	5.87E-03	5.37E-03
BP	GO:0140014	Mitotic Nuclear Division	10	5.87E-03	5.37E-03
BP	GO:0071103	Dna Conformation Change	9	4.11E-02	3.76E-02
BP	GO:0098813	Nuclear Chromosome Segregation	8	4.11E-02	3.76E-02
BP	GO:0000819	Sister Chromatid Segregation	8	1.70E-02	1.55E-02
BP	GO:0000070	Mitotic Sister Chromatid Segregation	8	5.87E-03	5.37E-03
CC	GO:0098687	Chromosomal Region	12	5.09E-04	4.71E-04
CC	GO:0005819	Spindle	9	1.62E-02	1.50E-02
CC	GO:0000775	Chromosome, Centromeric Region	9	5.09E-04	4.71E-04
CC	GO:0000793	Condensed Chromosome	7	2.09E-02	1.94E-02
CC	GO:0000776	Kinetochore	7	2.15E-03	1.99E-03
CC	GO:0000779	Condensed Chromosome, Centromeric Region	6	5.61E-03	5.20E-03
CC	GO:0000777	Condensed Chromosome Kinetochore	6	3.66E-03	3.39E-03
CC	GO:0072686	Mitotic Spindle	5	2.17E-02	2.01E-02

P-value[1] was adjusted by the Benjamini-Hochberg procedure.

Abbreviation: MSS-EO-CRC, early-onset colorectal cancer with microsatellite stable; GO, Gene Ontology; BP, Biological Process; CC, Cellular Components; FDR, False Discovery Rate.

Supplementary Table 4. Clinical characteristics of GEO and TCGA cohorts used for the development of the prognostic model.

Variables	GEO (N = 62)	TCGA (N = 33)
Age, year[1]	41.52 ± 6.38	43.94 ± 4.79
Gender		
Female	41 (66.00%)	18 (55.00%)
Male	21 (34.00%)	15 (45.00%)
Tumor Stage (AJCC 7th)		
I&II	27 (44.00%)	14 (42.00%)
III&IV	35 (56.00%)	19 (57.00%)

Abbreviations: AJCC, American Joint Committee on Cancer.

Data were represented as n (%) unless otherwise annotated. Age[1] was presented as mean ± standard deviation.

Supplementary Table 5. Identification of genes associated with the prognosis of MSS-EO-CRC patients.

Genes	Hazard ratio	95% Confidence interval	*P*-value
RPL7	6.877	0.2553-185.2283	0.251
**LYN**	0.028	0.0001-4.8309	**0.173**
POP1	0.931	0.0429-20.1837	0.964
IGDCC4	3.722	0.3175-43.6298	0.295
DKK1	1.506	0.4872-4.6565	0.477
NOM1	0.076	0.0007-7.9264	0.277
PARP1	0.122	0.0006-24.5372	0.437
MTHFD1	0.626	0.0004-922.8145	0.900
**PAK6**	0.063	0.0017-2.3561	**0.135**
**EIF3B**	0.007	0.0000-4.1048	**0.127**
**MCM7**	0.008	0.0001-0.5582	**0.026**
CHSY3	2.898	0.3204-26.2013	0.344
**TMEM39B**	0.047	0.0005-3.9815	**0.177**
TMEM41A	1.990	0.0105-374.4286	0.797
NCAPH	0.268	0.0058-12.3502	0.501
SMARCC2	1.432	0.0632-32.419	0.822
**MB21D2**	82.748	0.5466-12525.3747	**0.085**
**OTUD6B**	15.515	0.3588-670.8516	**0.154**
GAL	0.846	0.1336-5.3567	0.859
ZNF850	2.716	0.1844-39.9907	0.467
**SLC9B2**	24.195	1.2137-482.279	**0.037**
**CPLX1**	0.052	0.0043-0.6307	**0.020**
TRIM28	0.120	0.0006-21.5253	0.423
**KCNJ8**	19.226	0.839-440.5277	**0.064**
SLC28A3	1.201	0.2765-5.2184	0.807
NEK2	0.605	0.0191-19.0636	0.775
**WDR5**	0.006	0.0000-1.6840	**0.075**
TFAP2C	2.748	0.1171-64.4895	0.530
LMNB1	0.601	0.0039-90.2421	0.842
**YDJC**	0.013	0.0000-2.8762	**0.115**
**DDX20**	46.789	0.3999-5474.2078	**0.113**
ESPL1	0.174	0.0022-13.6901	0.433
RUVBL1	5.411	0.0831-352.1736	0.428
URB2	0.030	0.0000-11.8718	0.251
BMP7	1.666	0.0785-35.3518	0.743
PDIA5	106.134	0.0806-139740.9125	0.203
RAB15	0.135	0.0004-36.4832	0.483
MTHFD2	1.861	0.0396-87.4233	0.752

MCM3	0.431	0.0017-104.2334	0.764
**GRINA**	0.017	0.0000-4.0844	**0.145**
BYSL	1.688	0.0160-177.8164	0.826
**SKA1**	0.108	0.0069-1.6634	**0.111**
**REEP6**	0.097	0.0050-1.8628	**0.122**
**MMP19**	53.412	1.8410-1549.5156	**0.021**
DNMT1	0.166	0.0017-15.7989	0.439
MICB	0.606	0.0500-7.3474	0.694
C1orf216	3.225	0.0538-193.1411	0.575
IMPDH2	0.295	0.0020-41.5224	0.628
BOK	0.449	0.0223-9.0431	0.601
NDC80	1.395	0.1235-15.7481	0.788
CDCA8	0.246	0.0042-14.3655	0.500
PTDSS1	0.052	0.0001-17.3743	0.319
GAS2	0.622	0.1355-2.8514	0.541
TTC7B	3.052	0.2456-37.9307	0.385
HAUS6	8.106	0.2911-225.6939	0.218
**THEM6**	0.026	0.0006-1.0519	**0.053**
EGFL6	2.270	0.2916-17.675	0.434
PCED1B	1.073	0.0873-13.1804	0.956
HJURP	4.706	0.0523-423.3279	0.500
**GPR137B**	9.551	0.3576-255.0345	**0.178**
EIF3C	0.760	0.0500-11.5566	0.844
LAPTM4B	0.246	0.0025-24.0476	0.548
TMEM147	0.030	0.0000-15.7206	0.272
IGFBP2	0.308	0.0423-2.2368	0.244
**NUDCD1**	26.679	0.6131-1160.7114	**0.088**
GALNT6	2.280	0.1335-38.9194	0.569
C2CD4A	1.369	0.2889-6.4867	0.692
TTK	4.933	0.1179-206.3585	0.402
**CHN1**	56.764	1.1226-2870.0637	**0.044**
DCUN1D5	1.297	0.0161-104.5438	0.907
RPP40	1.601	0.1334-19.2008	0.710
LBH	6.826	0.2038-228.5554	0.284
DTL	0.332	0.0108-10.1262	0.527
MELK	1.557	0.0647-37.4559	0.785
PUS7	0.057	0.0004-6.7149	0.239
CCDC113	0.763	0.1094-5.3176	0.785
HPRT1	0.080	0.0003-19.7164	0.369
RAD54B	0.937	0.0251-34.886	0.972
ODC1	0.497	0.0054-45.1958	0.761
PSTPIP2	0.238	0.0109-5.1749	0.361

AHCY	0.321	0.0037-27.3828	0.616
MAD2L1	0.259	0.0074-9.0648	0.456
**WASF1**	53.376	2.3037-1236.6972	**0.013**
RACGAP1	0.293	0.0014-58.6414	0.650
SPIN4	1.507	0.0187-120.933	0.854
LPCAT1	1.754	0.0557-55.2411	0.749
MMP7	1.304	0.1596-10.6441	0.805
DDIAS	0.350	0.0098-12.4218	0.564
ANLN	0.437	0.0124-15.2807	0.648
LOXL1	5.037	0.3442-73.7045	0.238
OSR2	0.399	0.0534-2.9733	0.370
DEFA6	0.877	0.2636-2.9162	0.830
CENPW	0.136	0.0025-7.3527	0.327
TRIP6	2.688	0.196-36.8676	0.459
**LIPG**	0.147	0.0178-1.2125	**0.075**
GPT	0.094	0.0001-73.2295	0.486
**EPS8L2**	0.000	0.0000-0.8792	**0.046**
**GPER1**	0.001	0.0000-0.7234	**0.040**
SH3D19	1.849	0.0232-147.0316	0.783
CD58	1.825	0.0106-311.681	0.819
CAST	3.498	0.0078-1564.4759	0.688
CCDC186	10.484	0.149-737.4458	0.279
EHF	0.797	0.0272-23.3677	0.896
GLRX	0.054	0.0000-58.0754	0.413
SARAF	9.115	0.063-1318.4455	0.384
**STAM2**	135.776	0.5067-36380.7431	**0.085**
**TNFRSF14**	0.000	0.0000-0.0056	**0.001**
SLC9A2	0.190	0.0117-3.0818	0.243
**CAMK2N1**	0.014	0.0004-0.4333	**0.015**
**ENTPD5**	0.000	0.0000-0.0467	**0.003**
RSRP1	4.168	0.0241-719.7112	0.587
FEM1C	6.585	0.0379-1143.9629	0.474
RMDN2	1.600	0.0125-203.2498	0.849
LYRM7	3.021	0.0728-125.2226	0.561
STAP2	0.115	0.0037-3.5572	0.217
SELENBP1	0.306	0.0123-7.5984	0.470
GK	3.572	0.0564-226.1018	0.547
CDHR2	0.130	0.0029-5.6259	0.288
SULT1B1	0.582	0.0204-16.5827	0.752
RASSF6	0.988	0.0782-12.4764	0.992
PSD3	5.010	0.1823-137.6313	0.340
DPP10	1.421	0.0719-28.0616	0.817

CDHR5	0.238	0.0129-4.3491	0.333
NIPAL1	0.246	0.0178-3.3918	0.295
SLC16A1	0.959	0.0469-19.5598	0.978

The genes with the bold value represented a *P*-value less than 0.200 in the univariate Cox regression.

Supplementary Table 6. Univariable and multivariable Cox regression to identify independent prognostic factors in the GEO cohort.

**Characteristics**		**Overall survival in GEO Cohort**			
	Number of patients	Univariable Cox analysis		Multivariable Cox analysis	
		HR (95% CI)	*P*-value	HR (95% CI)	*P*-value
Age	62	1.03 (0.95-1.11)	0.505	——	——
Gender (Male vs Female)	62	1.7 (0.71-4.06)	0.232	——	——
Tumor Stage (I, II, III or IV)	62	2.01 (1.23-3.29)	**0.005**	2.05 (1.24-3.38)	**0.005**
Risk Score	62	1.28 (1.05-1.56)	**0.013**	1.32 (1.06-1.63)	**0.013**

The bold values were less than 0.05.

Abbreviation: HR, hazard ratio; CI, confidence interval.

Supplementary Table 7. Detailed information of included GEO datasets.

Variables	GSE39582	GSE39084[1]	GSE9348	GSE170999	GSE18088	GSE75316
PMID	23700391	25083765	20143136	33817986	21465190	27354468
Platform	GPL570 (Affymetrix Human Genome U133 Plus 2.0 Array)					
Age (years)						
>= 50	517	42	82	56	46	55
< 50	67	20	0	20	7	4
Gender						
Male	322	31	46	43	26	41
Female	263	31	36	33	27	18
MSI status						
MSI-High/Low	75	11	0	0	19	11
MSS	444	51	70	76	34	48
TNM stage						
I&II	297	26	70	15	53	30
III&IV	265	35	0	61	0	29
Number of samples						
CRC	566	62	70	76	53	59
Normal	19	0	12	0	0	0
OS status						
Alive	371	36	——	——	——	——
Death	191	26	——	——	——	——
RFS status						
Non-recurrence	380	——	——	——	——	——
Recurrence	177	——	——	——	——	——

Abbreviation: MSI, microsatellite instability; MSS, microsatellite stable; TNM, Tumor-Node-Metastasis; OS, overall survival; RFS, recurrence-free survival. [1]: exclude patients with Lynch syndrome.

Supplementary Figures and Corresponding Legends:

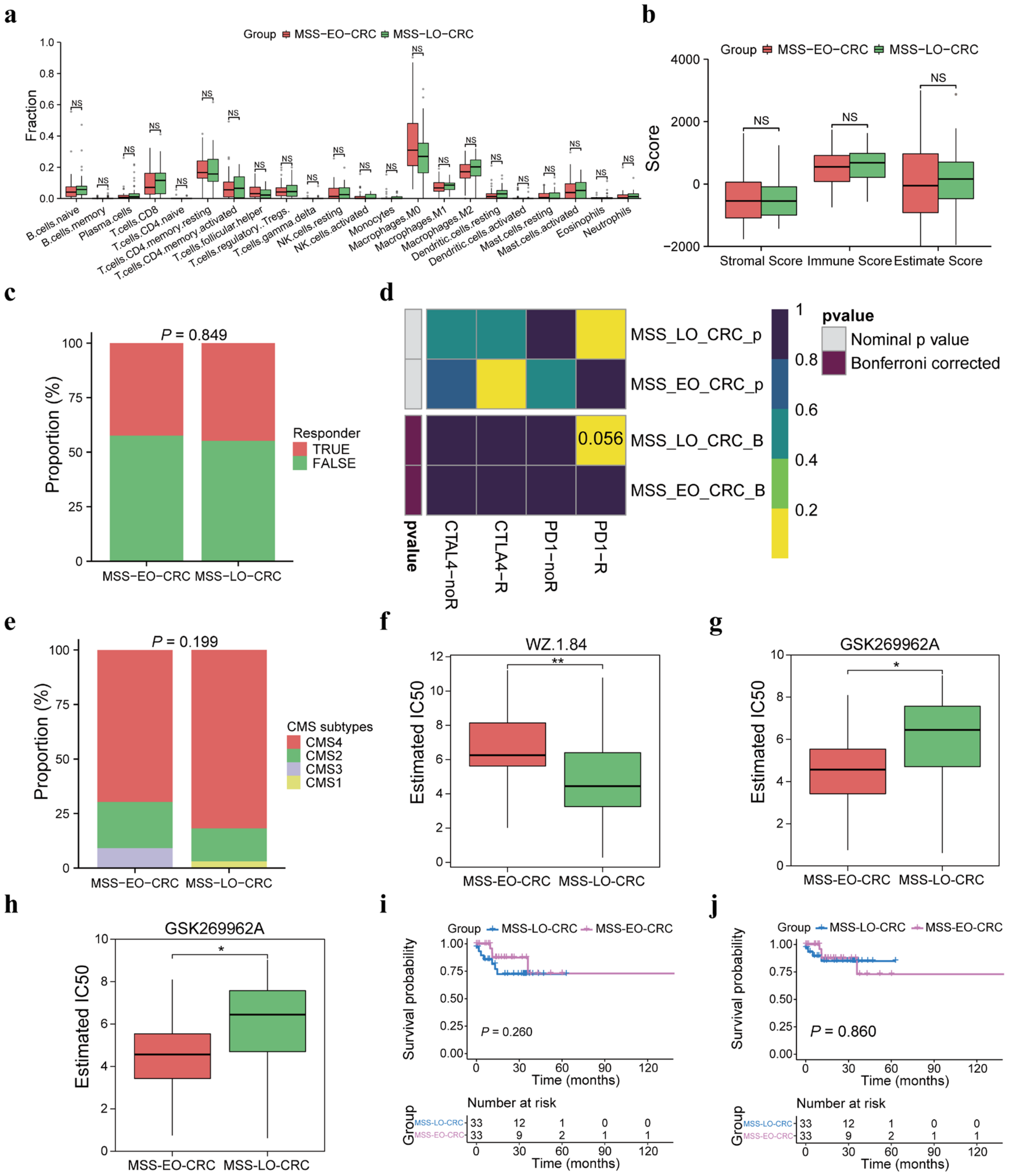

Supplementary Figure 1. Characterization of the tumor microenvironment and prognosis of early-onset CRC with MSS from TCGA cohort.

a) Comparison of tumor-infiltrating immune cells between MSS-EO-CRC and MSS-LO-CRC. *P*-values were corrected using the Benjamini-Hochberg method. b) Comparison of the enrichment score between MSS-EO-CRC and MSS-LO-CRC. c, d) Comparison of Immunotherapeutic responses between MSS-EO-CRC and MSS-LO-CRC using TIDE and SubMap algorithm, respectively. e) Consensus molecular subtype

analysis of MSS-EO-CRC versus MSS-LO-CRC. f, g, h) Drug sensitivities comparison between MSS-EO-CRC and MSS-LO-CRC. i) Overall survival comparison between MSS-EO-CRC and MSS-LO-CRC. j) Disease-specific survival comparison between MSS-EO-CRC and MSS-LO-CRC. The log-rank test *P* values were shown for each Kaplan-Meier plot. *** $P < 0.001$; ** $P < 0.01$; * $P < 0.05$. Abbreviation: MSS-EO-CRC, early-onset colorectal cancer with microsatellite stable; MSS-LO-CRC, late-onset colorectal cancer with microsatellite stable; NS, no significance.

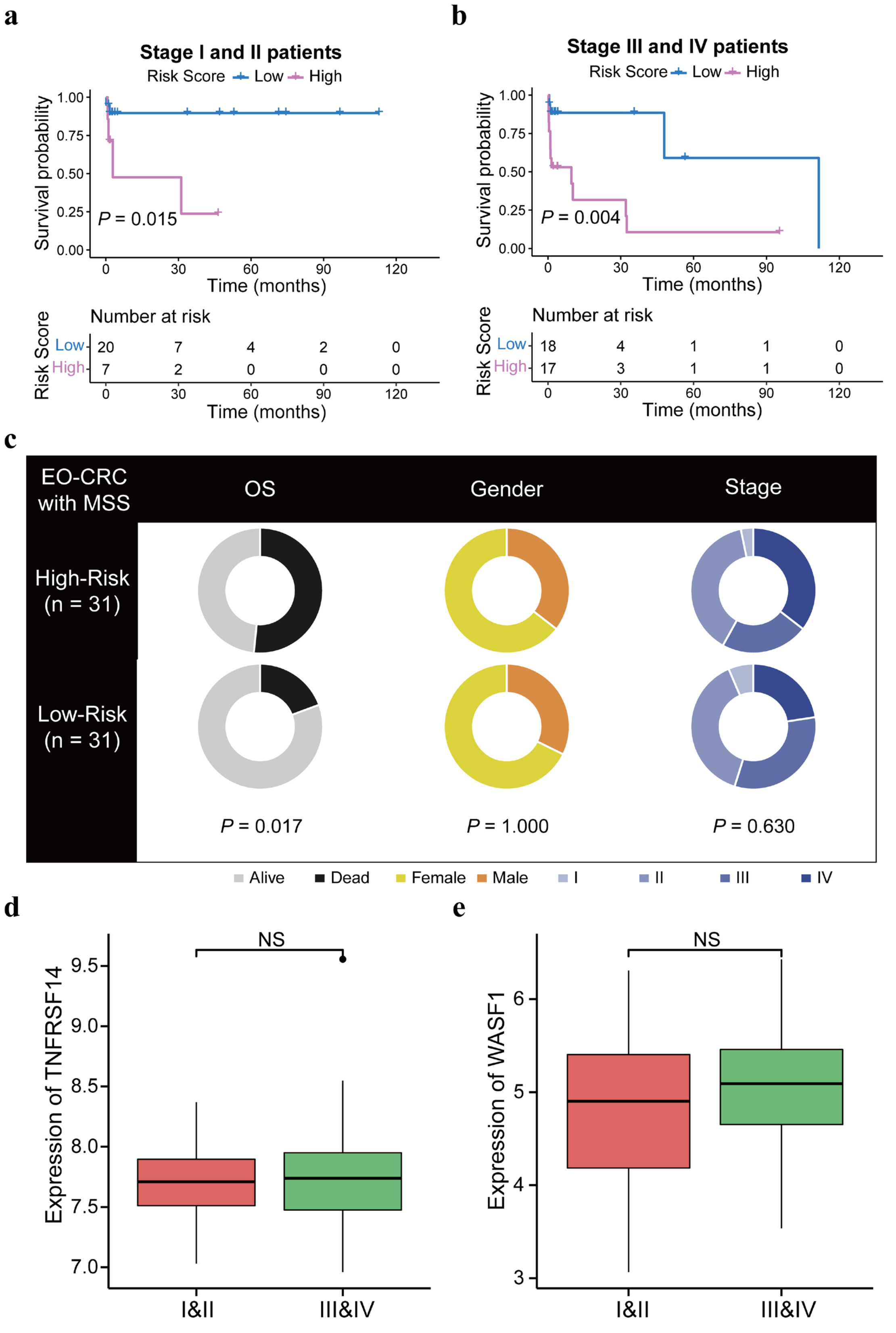

Supplementary Figure 2. Correlation analysis between clinical features and the risk score in MSS-EO-CRC cohort based on GEO datasets.

a, b) Kaplan-Meier plot of the risk score stratified by tumor stage. c) Characteristics of overall survival, gender, and tumor stage between high- and low-risk score groups. d, e) Expression differences of TNFRSF14 and WASF1 between early-stage and advanced MSS-EO-CRC, respectively. The log-rank test *P* values were shown for each Kaplan-Meier plot. *** $P < 0.001$; ** $P < 0.01$; * $P < 0.05$.

Abbreviation: MSS-EO-CRC, early-onset colorectal cancer with microsatellite stable; NS, no significance.

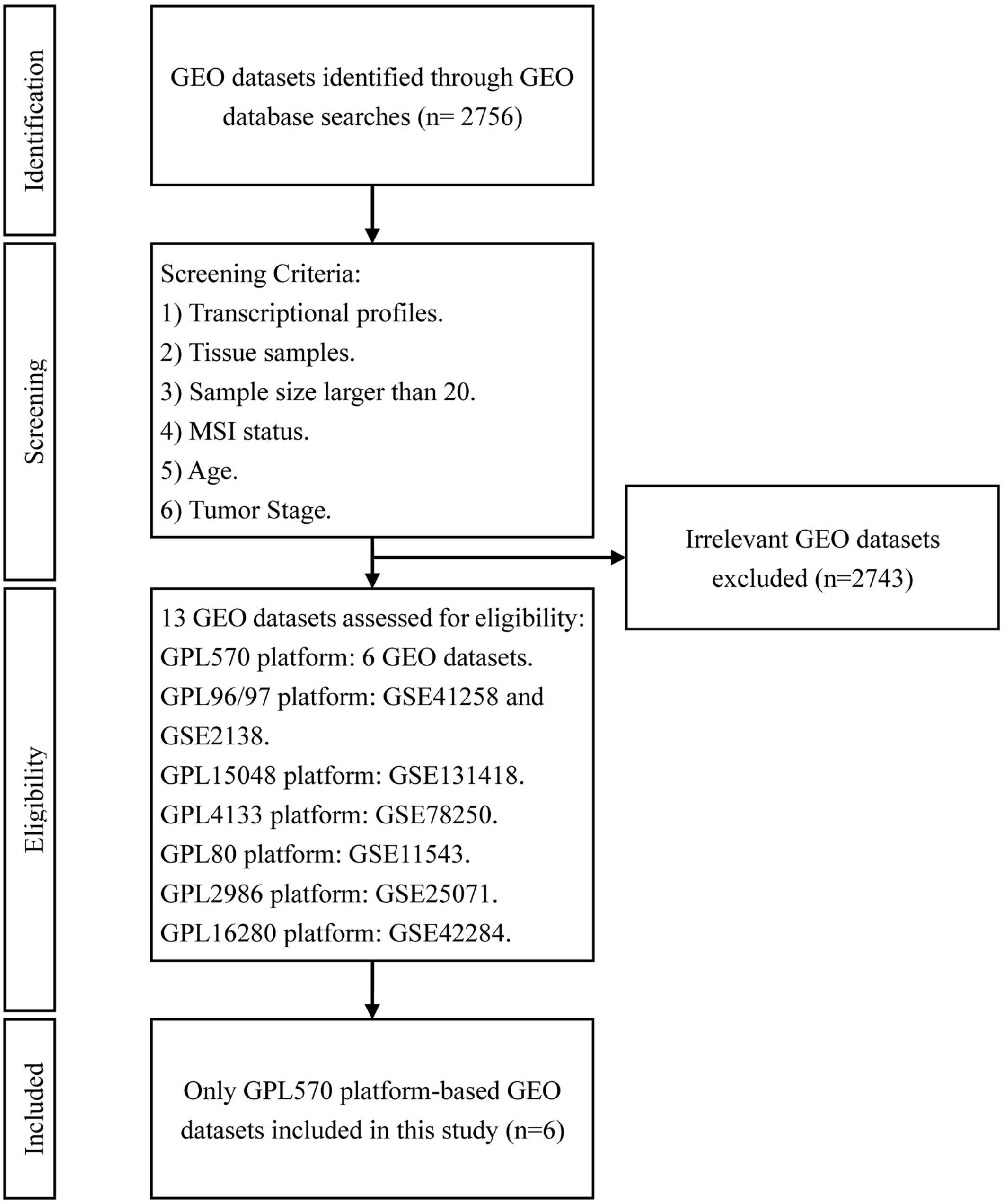

Supplementary Figure 3. Flow diagram of identifying and selecting eligible GEO datasets.

Abbreviation: GEO, Gene Expression Omnibus; MSI, microsatellite instability

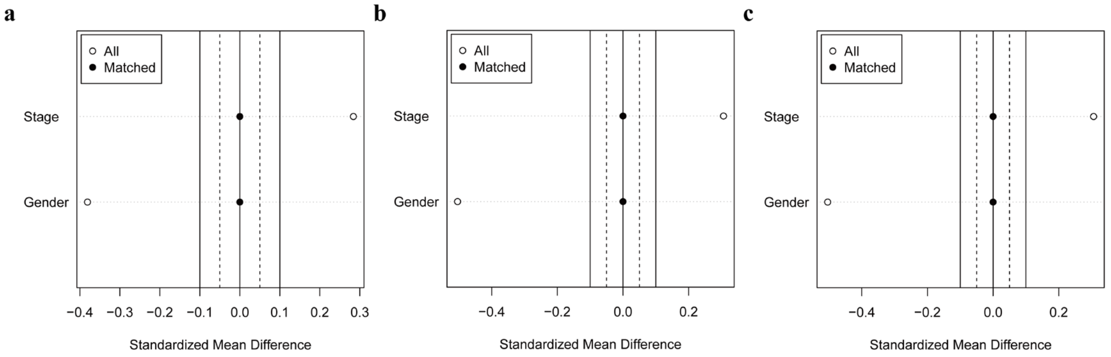

Supplementary Figure 4. Evaluation of the matching quality between MSS-EO-CRC and MSS-LO-CRC patients.

a) The assessment of matching quality between 88 MSS-EO-CRC and 88 MSS-LO-CRC patients. b) The assessment of matching quality between 62 MSS-EO-CRC and 62 MSS-LO-CRC patients for overall survival analysis. c) The assessment of matching quality between 43 MSS-EO-CRC and 43 MSS-LO-CRC patients for recurrence-free survival analysis.

Abbreviation: MSS-EO-CRC, early-onset colorectal cancer with microsatellite stable; MSS-LO-CRC, late-onset colorectal cancer with microsatellite stable.

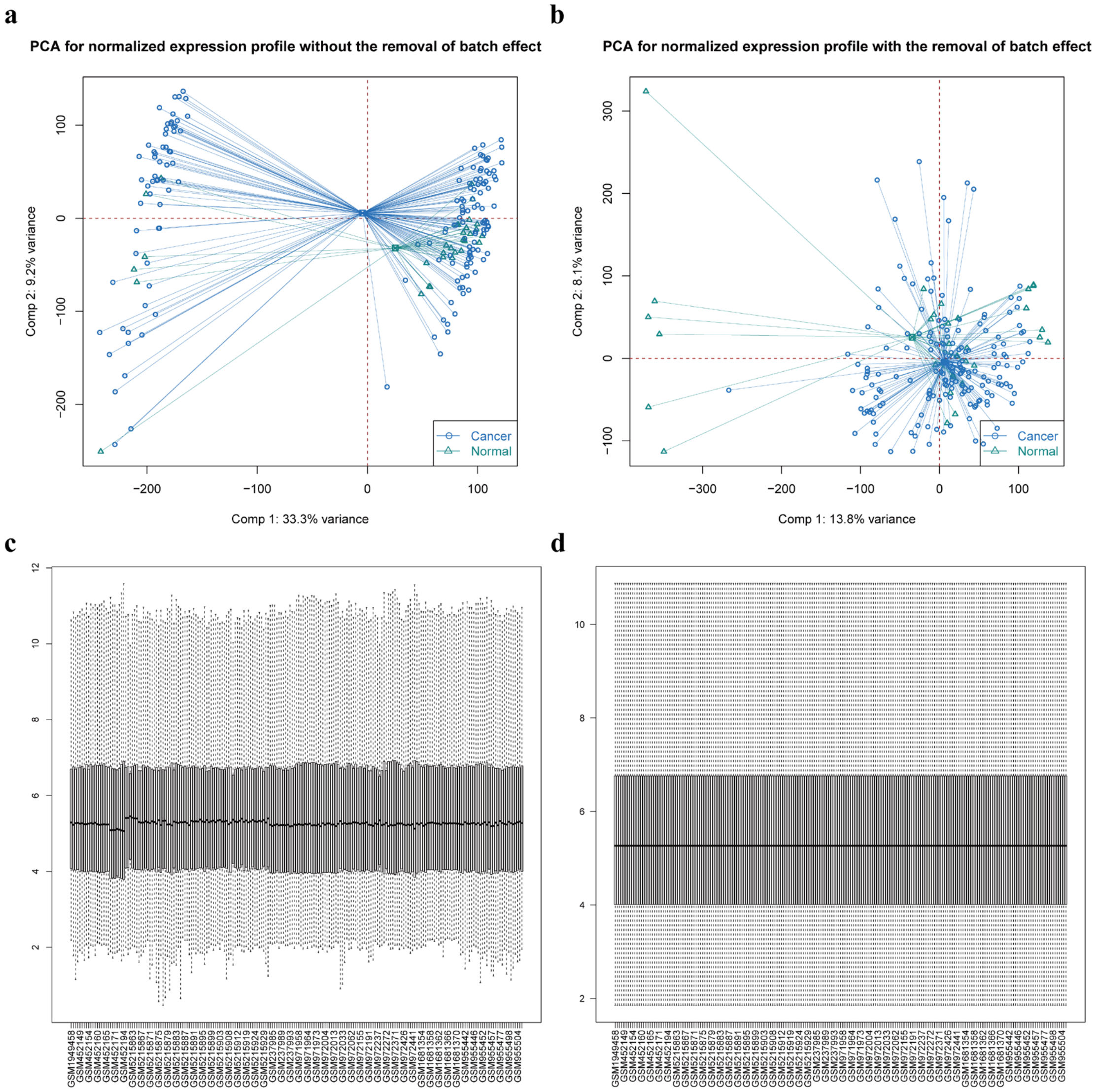

Supplementary Figure 5. The pre-processing of merged GEO datasets.

a) PCA graph of the merged MSS-CRC and normal samples without removing batch effects. b) PCA graph of the merged MSS-CRC and normal samples with the removal of batch effects. c) The boxplot of merged MSS-CRC and normal samples without quantile normalization. d) The boxplot of merged MSS-CRC and normal samples with quantile normalization.

Abbreviation: GEO, Gene Expression Omnibus; MSS-CRC, colorectal cancer with microsatellite stable.
